# Targeting the GRP78 Pathway for Cancer Therapy

**DOI:** 10.3389/fmed.2020.00351

**Published:** 2020-07-30

**Authors:** Guanhua Lu, Hui Luo, Xiao Zhu

**Affiliations:** ^1^Guangdong Key Laboratory for Research and Development of Natural Drugs, The Marine Biomedical Research Institute, Guangdong Medical University, Zhanjiang, China; ^2^The Marine Biomedical Research Institute of Guangdong Zhanjiang, Zhanjiang, China; ^3^Southern Marine Science and Engineering Guangdong Laboratory, Zhanjiang, China; ^4^The Key Lab of Zhanjiang for R&D Marine Microbial Resources in the Beibu Gulf Rim, Guangdong Medical University, Zhanjiang, China

**Keywords:** GRP78, endoplasmic reticulum stress, autophagy, apoptosis, tumor-targeted therapy

## Abstract

The 78-kDa glucose-regulated protein (GRP78) plays an important part in maintaining protein stability, regulating protein folding, and inducing apoptosis autophagy, which is considered as a powerful protein. Meanwhile, it also plays a role in ensuring the normal function of organs. In recent years, more and more researches have been carried out on the targeted therapy of GRP78, mainly focusing on its relevant role in tumor and its role as a major modulator and modulator of subordinate pathways. The ability of GRP78 to respond to endoplasmic reticulum stress (ERS) determines whether tumor cells survive and whether the changes in expression level of GRP78 regulated by endoplasmic reticulum (ER) caused by various factors will directly or indirectly affect cell proliferation, apoptosis, and injury, or reduce the body's defense ability, or have protective effects on various organs.

## Introduction

**Figure 1:**
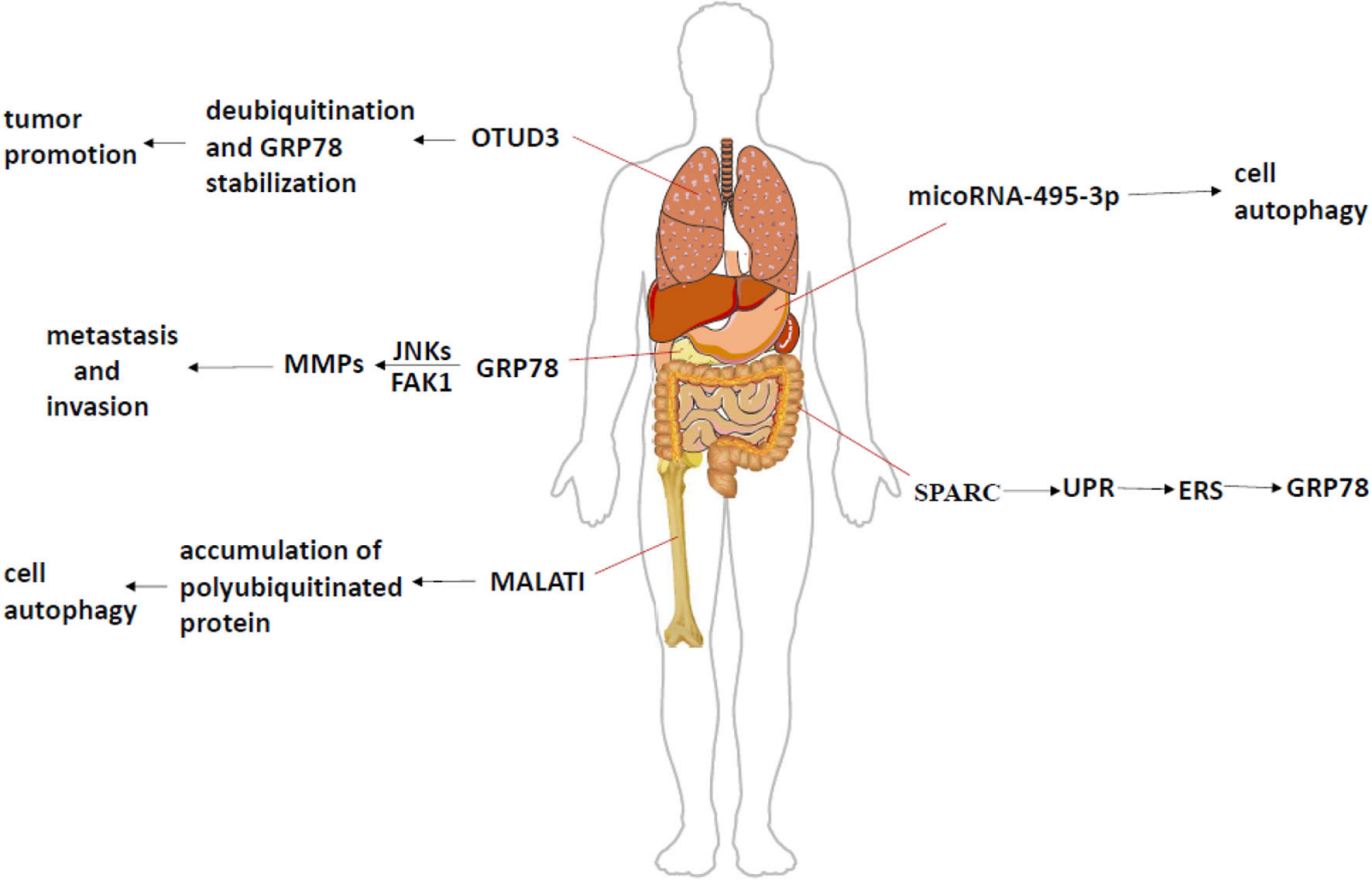
The mechanism of GRP78 in different tumors. In lung adenocarcinoma, GRP78, as a specific substrate of OTUD3, mediates deubiquitination under the state of high expression of OTUD3, and enables the stable proliferation of tumor cells. GRP78 activates JNKs and FAK1 pathways in pancreatic cancer, and then increases the expression of downstream products and promotes tumor invasion and metastasis. In the process of multiple myeloma, the expression of MALATI will lead to the accumulation of polyubiquitinated proteins, which will activate ERS, promote the expression of GRP78, and induce cell apoptosis. MicroRNA in gastric cancer leads to different malignant phenotypes through different effects on GRP78. SPARC can interfere ERS by affecting UPR in patients with colorectal cancer, thereby changing the expression of GRP78 and controlling the apoptosis of different cells.

Glucose regulated protein (GRP78) is a mature endoplasmic reticulum (ER)-resident chaperone, belonging to the large chaperone family of heat-shock protein 70 (HSP70) molecules (1). Physiologically, GRP78 binds to the polypeptide chain in a non-covalent bond on the ER and then disassociates, facilitating proper protein folding and assembly, helping protein transport across the ER (2). It has 60% homology with the HSP70 family, and its substrate binding region contains a lysine-rich structural domain (3), which has the characteristic of binding to abnormal proteins under stress conditions, but it has differences in the regulation of protein expression (4). In ER, GRP78 assists the ER protein to stabilize and induces protein folding reaction (unfolded protein response, UPR). The surface-related GRP78 plays a role in cell protection and mediated cytoskeletal remodeling (5), while the secreted GRP78 plays an immunomodulatory role (6). GRP78 can move in under the condition of ER outside the region, even secrete to the outside of the cell with the corresponding ligand binding, playing its function by affecting various signaling pathways, influencing the cell signal transduction and the development (Figure 1). Under certain pathological conditions, cytokines produced by the body, due to exposure to inflammatory environment, promote the surface translocation of GRP78, which further explains the prominent position of GRP78 in many diseases (7). Recent studies have demonstrated that GRP78 has far more functions than this (4, 8, 9). A series of researches have shown that GRP78 plays a valid role in tumor activity and treatment.

## Correlation With Tumor Occurrence and Development

**Figure 2:**
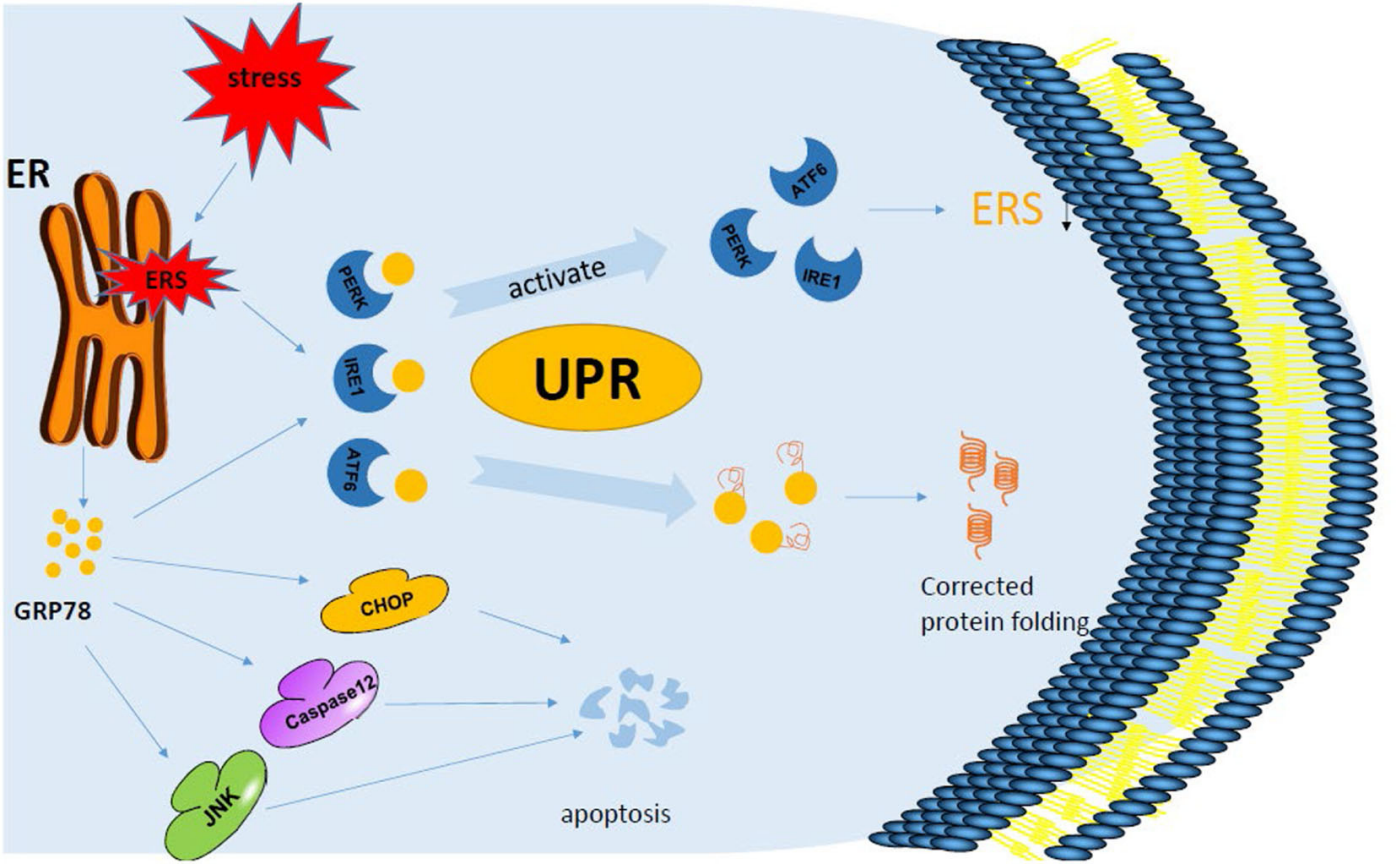
GRP78 and endoplasmic reticulum stress. When a cell is under the influence of foreign factors, the endoplasmic reticulum is stressed, leading to the appearance of unfolded proteins or misfolded proteins inside the cell, which accumulates in the cell. At this time, three specific signaling pathways, PERK, IRE1, and ATF6, trigger the unfolded protein response. PERK, IRE1, ATF6, and GRP78 are activated from the binding state to the detached state. GRP78 binds to unfolded proteins or misfolded proteins to correct protein folding, while PERK and other physiological reactions reduce protein synthesis after a series of phosphorylation. In addition, the expression of GRP78 is up-regulated when ERS occurred, and the pro-apoptotic pathways caspase 12, JNK, and CHOP are activated. When UPR exceeds a specified limit, it also induces apoptosis through the caspase 12 pathway.

Advanced breast cancer patients have a high mortality rate, mainly because of tumor metastasis. GRP78 has a low expression in benign breast lesions, but it greatly increases in breast cancer (10–12). Stathmin1 (STMN1), also known as oncoprotein 18, is a phosphorylated protein associated with multiple tumor metastases, which can bind with GRP78 to form STMN1-GRP78 stable complex, mediating tumor metastasis (13) (Table 1). High expression of GRP78 can promote the expression of MMPs (matrix metalloproteinases) and metastasis and invasion of pancreatic cancer by activating some pathways like JNK (c-Jun N-terminal kinase) and FAK (focal adhesion kinase) (24) (Figure 1). However, the deletion of GRP78 not only reduced the expression of MMPs but also inhibited the RhoA (Ras homolog gene family, member A) signaling pathway and prevented tumor invasion (25). CRIPTO (Teratocarcinoma-derived growth factor 1), one of the major regulators, participate in the development of prostate cancer. It has been found that its expression is elevated to many cancers, especially in metastatic tumors, and GRP78 also shows high expression (Figure 1) (11, 26, 27). As we all know, ERS in tumor cells is an important tumor response to help tumor survival. The knockout of CRIPTO or GRP78 can reduce the invasion of cancer cells, thereby reducing cell proliferation, migration, colony formation, and other processes (28). KRAS (Kirsten rat sarcoma viral oncogene) mutations are present in 90% of pancreatic cancer patients, but an expression of only half of GRP78 prevents early pancreatic cancer development (29). Therefore, these results approve the importance of GRP78 in the metastasis and survival of most tumors whatever directly or indirectly. In colorectal cancer, SPARC (Secreted Protein, Acidic and Rich in Cysteine) interference ERS to further enhance apoptosis by regulating the UPR (Figure 2); the low expression of SPARC and GRP78 prompts settlement colorectal cancer patients with a lower survival rate (Figure 1; Table 1) (14). In addition, hepatocellular carcinoma cells under ER stress transfer specific miRNA through exosomes to infiltrating macrophages in the tumor micro-environment to promote the immune escape of hepatocellular carcinoma cells (30–33). As we all know, the diversity of various binding proteins and functional results of GRP78 reflects the function of GRP78 in regulating cell activities and making different responses to ER, so as to make the body change, aggravate damage, or play a protective role.

**Table 1:** The specific mechanisms of cancer and therapy drugs.

**Types**	**Signaling molecules**	**Therapeutic mechanism**
**Cancer**		
Breast cancer (13)	STMN1	Reduced the expression of MMPs, inhibit the RhoA signaling pathway
Colorectal cancer (14)	SPARC	Regulate the UPR and enhance apoptosis
Head and neck cancer (15)	MUL1	Inhibit the ubiquitination of MUL1
Multiple myeloma (16)	MALAT1	Induce ERS to up-regulate ER sensor protein expression, and activate autophagy
Lung adenocarcinoma (17)	OTUD3	Inhibit tumor cell deubiquitination and decrease protein stability
Gastric cancer (18)	mir-495-3p	Induce autophagy
**Drugs**		
Ferritin probe (19, 20)	SP94	Binding to GRP78 mediates endocytosis into lysosomes and ferritin cleavage releases doxorucin
OSU-03012/ phosphodiesterase 5 inhibitor (21)		Decreased expression of viral and oncogene receptors
Thiazolaminobenzene sulfamides (22)		Strengthen the ERS, inducing autophagy and apoptosis
Triptolide (23)		Induce chronic ERS, down-regulate the GRP78 expression

## New Targets for Tumor Therapy

GRP78 can promote the survival of head and neck cancer cells by maintaining lysosomal activity (Figure 1), meanwhile, through inhibiting the development of head and neck cancer by inducing a series of ubiquitination of mitochondrial ubiquitin ligase activator (such as MUL1, one of E3 ubiquitin-protein ligases) to down-regulate GRP78 (Table 1) (15). Tumor growth and GRP78 expression were inhibited after MUL1 cancer cell was knocked out, suggesting that the MUL1–GRP78 axis will become a new strategy for the treatment of head and neck cancer. In multiple myeloma, the antagonistic effect of a form of RNA called MALAT1 (metastasis-associated lung adenocarcinoma transcript1) will lead to the accumulation of ubiquitin proteins, induce ERS, up-regulate the expression of ER sensor proteins such as GRP78, activate autophagy, and induce apoptosis (Figure 1; Table 1) (16, 34). This suggests that the key link in the treatment of myeloma may be to induce ERS and promote the up-regulation of GRP78 expression to control the tumor. A domain-containing protein 3 (OTUD3) showed high expression in lung adenocarcinoma, and GRP78 could be used as a specific substrate. OTUD3 was first identified as the deubiquitination enzyme of GRP78, mediating deubiquitination in tumor cells and maintaining the function of GRP78, so as to stabilize the proliferation of tumor cells (Figure 1; Table 1) (17). In patients with gastric cancer, microRNA mir-495-3p regulates autophagy by targeting GRP78. The expression level of GRP78 can antagonize the autophagy induced by mir-495-3p. Clinically, both the up-regulation of GRP78 and the down-regulation of mir-495-3p are linked to the malignant phenotype of gastric cancer (Figure 1; Table 1) (18). Therefore, GRP78 can be combined with the specific factor as an indicator of treatment response and prognostic indicator (35, 36).

Recently, researchers have designed a new ferritin probe that can specifically identify and kill liver cancer cells. The targeted peptide SP94 on its surface can specifically bind GRP78 on the surface of liver cancer cells to identify liver cancer (19, 20). GRP78 further mediates endocytosis into lysosomes of liver cancer cells, and ferritin lysis releases adriamycin under acidic environment, so as to kill liver cancer cells (Table 1) (19, 20). In addition, GRP78 level on the surface of cancer cells has been found in prostate cancer studies to be related to tumor stage, and the level of anti-GRP78 antibody is parallel to the concentration of tumor-specific antigen in serum (27, 37, 38), which further suggests the effect of GRP78 on driving tumor progression. Therefore, the expression of GRP78 can be considered as a promoter of many cancer characteristics, and it has been proved that it is up-regulated not only in a variety of tumor cells, but also in tumor-related macrophages. Under the action of ERS, multiple factors combined with GRP78 work together to regulate the autophagy apoptosis of cells and make the cells progress in different directions (Figure 2).

## Tumor Effect of Drugs and Improvement of Drug Resistance

As the treatment progresses, cancer cells will develop resistance to therapeutic drugs. There are studies that have shown that high levels of GRP78 in tumor cells lead to multidrug resistance (MDR) in treatment (39); thus, GRP78 can be a good target. Controlling its expression can restore the sensitivity of tumor cells to therapeutic drugs (40–43). In addition, OSU-03012 and phosphodiesterase 5 inhibitor were used in some experiments to target GRP78 and its related proteins, which can prevent some viruses and bacteria from replicating and killing most tumor cells without harming other normal cells (Table 1) (21). This mechanism is mainly achieved by reducing the expression of virus receptor and oncogene receptor. Thiazolaminobenzene sulfamides can bind to GRP78 in the ER of melanoma cells and enhance ERS, exert cytotoxic effects, and induce autophagy and apoptosis and other anti-tumor effects (Figure 2); in addition, it can reverse the sensitivity of drug-resistant mice to anti-tumor drugs (Table 1) (22). In traditional Chinese medicine, triptolide, an extract of *Tripterygium wilfordii*, can induce chronic ERS and down-regulate the expression of GRP78, leading to cell death (Figure 2; Table 1) (23). Therefore, the treatment of a tumor can be started from the proliferation and growth of tumor cells and the use of the drug inhibition or targeting GRP78 to control its expression and improve the sensitivity to treatment.

## Participation in Immune Activation and Immunosuppression

All members of the HSP70 family have the ability to bind to tumor-specific antigens and mediate immune responses. As a member with a certain correlation with antigen epitopes, GRP78 mainly stimulates anti-tumor immune responses through the cross-expression of histocompatibility complex molecules and initiates CD8^+^ cytotoxic T cell responses (44). At present, radiation therapy has become one of the main methods to treat tumors. Cells irradiated by radiation will release damage-associated molecular patterns (DAMPs) by activating GRP78, and tumor cells will be devoured by antigen-presenting cells. At the same time, cytotoxic T cells will get information and promote the destruction of tumor cells (45–49). However, its overexpression on proliferating and quiescent cancer cells and tumor-related endothelial cells is conducive to escape from chemotherapy and other therapeutic methods. Meanwhile, it inhibits apoptosis and stimulates autophagy through a series of signaling pathways, thus reducing the sensitivity of tumor cells to radiotherapy and chemotherapy (50, 51). In addition, the anti-GRP78 antibody has been found to have anti-tumor activity, which can not only reduce the tumor growth rate but also enhance the efficacy of radiotherapy for non-small cell lung cancer (52, 53). This provides a more effective and feasible method for the limitation of internal medicine treatment of non-small cell lung cancer (54). Meanwhile, GRP78 can be used as a medium to induce the transformation of cell activities, so that the tumor cells are restricted or even die, which also provides a new path for physical-immune combination therapy to deal with some of the tumors that cannot be treated by non-surgical treatment.

## Perspectives

With the development of research and technology, it is believed that GRP78 may have obvious therapeutic potential as a target (55, 56). Both GRP78 itself and other forms of GRP78 have a unique role in controlling cell survival and signal transduction, which is developing into an excellent treatment tool for malignant tumors in the targeted treatment of some cancers, and is expected to be applied to other diseases. According to the biological characteristics of GRP78 in different tumors and the effects of its regulated downstream pathways, we can predict the positive or negative effects. Although the effect of GRP78 in the treatment of tumor is still not completely clear, it can be further studied according to its biological effects and characteristics, or combined with specific markers of diseases, which can provide certain theoretical guidance for precise treatment and achieve the goal of implementing treatment at the molecular level. Therefore, in view of the significance of GRP78 in the occurrence, development, and treatment of different diseases, targeted GRP78 has important value, and its mechanisms of action should be comprehensively considered, and further attention and in-depth research should be made on it.

## Author Contributions

GL and XZ performed the literature search and wrote the first draft of the manuscript. GL, HL, and XZ revised and edited the final version of the manuscript. All authors contributed to the article and approved the submitted version.

## Conflict of Interest

The authors declare that the research was conducted in the absence of any commercial or financial relationships that could be construed as a potential conflict of interest.

## Abbreviations

ANF: atrial sodium factor
ATF6: Activating transcription factor 6
CHOP: CCAAT/enhancer-binding protein homologous protein
CRIPTO: Teratocarcinoma-derived growth factor 1
DAMP: damage associated molecular patterns
ERS: endoplasmic reticulum stress
FAK: Focal adhesion kinase
GRP78: Glucose regulated protein
GATA-4: gata binding protein 4
HSP70
heat shock protein 70
IRE1: Inositol requiring enzyme
JNK: c-Jun N-terminal kinase
KRAS: Kirsten rat sarcoma viral oncogene
LXR: liver X receptor
MALATI: metastasis associated lung adenocarcinoma transcript1
MMPs: matrix metalloproteinases
OTUD3: Domain-containing protein 3
PERK: Protein kinase-like ER kinase
PI3K: Phosphoinositide 3-kinase
RhoA, Ras homolog gene family: member A
SPARC, Secreted Protein: Acidic and Rich in Cysteine
STMN1: Stathmin1
UPR: unfolded protein response.
